# Predictors of treatment response in a lupus nephritis population: lessons from the Aspreva Lupus Management Study (ALMS) trial

**DOI:** 10.1136/lupus-2021-000584

**Published:** 2022-05-30

**Authors:** Stephen McDonald, Sean Yiu, Li Su, Caroline Gordon, Matt Truman, Laura Lisk, Neil Solomons, Ian N Bruce, for the MASTERPLANS Consortium, Ian N Bruce

**Affiliations:** 1 The Kellgren Centre for Rheumatology, NIHR Manchester Biomedical Research Centre, Manchester University Hospitals NHS Foundation Trust, Manchester Academic Health Science Centre, Manchester, UK; 2 MRC Biostatistics Unit, School of Clinical Medicine, University of Cambridge, Cambridge, UK; 3 Rheumatology Research Group, Institute of Inflammation and Ageing, University of Birmingham, Birmingham, UK; 4 Aurinia Pharmaceuticals Inc, Victoria, British Columbia, Canada; 5 Centre for Epidemiology Versus Arthritis, Faculty of Biology, Medicine and Health, The University of Manchester, Manchester Academic Health Science Centre, Manchester, UK

**Keywords:** cyclophosphamide, treatment, lupus nephritis

## Abstract

**Objectives:**

To identify predictors of overall lupus and lupus nephritis (LN) responses in patients with LN.

**Methods:**

Data from the Aspreva Lupus Management Study (ALMS) trial cohort was used to identify baseline predictors of response at 6 months. Endpoints were major clinical response (MCR), improvement, complete renal response (CRR) and partial renal response (PRR). Univariate and multivariate logistic regressions with least absolute shrinkage and selection operator (LASSO) and cross-validation in randomly split samples were utilised. Predictors were ranked by the percentage of times selected by LASSO and prediction performance was assessed by the area under the receiver operating characteristics (AUROC) curve.

**Results:**

We studied 370 patients in the ALMS induction trial. Improvement at 6 months was associated with older age (OR=1.03 (95% CI: 1.01 to 1.05) per year), normal haemoglobin (1.85 (1.16 to 2.95) vs low haemoglobin), active lupus (British Isles Lupus Assessment Group A or B) in haematological and mucocutaneous domains (0.61 (0.39 to 0.97) and 0.50 (0.31 to 0.81)), baseline damage (SDI>1 vs =0) (0.38 (0.16 to 0.91)) and 24-hour urine protein (0.63 (0.50 to 0.80)). LN duration 2–4 years (0.43 (0.19 to 0.97) vs <1 year) and 24-hour urine protein (0.63 (0.45 to 0.89)) were negative predictors of CRR. LN duration 2–4 years (0.45 (0.24 to 0.83) vs <1 year) negatively predicted PRR. The AUROCs of models for improvement, CRR and PRR were 0.56, 0.55 and 0.51 respectively.

**Conclusions:**

Baseline variables predicted 6-month outcomes in patients with SLE. While the modest performance of models emphasises the need for new biomarkers to advance this field, the factors identified can help identify those patients who may require novel treatment strategies.

**WHAT IS ALREADY KNOWN ON THIS TOPIC**

- Lupus nephritis (LN) occurs in up to 60% of patients with SLE and is associated with significant morbidity and mortality.

**WHAT THIS STUDY ADDS**

- Longer duration of LN and higher proteinuria were associated with poorer renal response.
- Active non-renal disease and baseline damage were associated with poorer overall SLE responses.

**HOW THIS STUDY MIGHT AFFECT RESEARCH, PRACTICE AND/OR POLICY**

- Attention to these factors may improve trial stratification and identify patients in which to consider more novel treatment strategies.

## Introduction

Lupus nephritis (LN) occurs in up to 60% of patients with SLE and is associated with significant morbidity and mortality.^1 2^ Up to 15% will progress to end-stage renal failure after 10 years.^3^ Treatments consist mainly of immunosuppressive drugs, with slow response, modest efficacy and significant side effects. There is a need to develop better early predictors of overall response and renal response in patients with SLE. Developing a more personalised approach to treatment may help mitigate longer-term complications.^4^

Clinical factors such as proteinuria and serum creatinine 1 year after starting treatment for LN have been demonstrated to be predictors of long-term renal response.^5 6^ High baseline serum creatinine, failure to achieve remission, hypertension and nephritic flares have also been associated with poor renal outcome.^7^ Demographic factors such as increasing age and male gender,^8^ as well as baseline histological findings, such as increased chronicity index and interstitial fibrosis, are all markers of worse renal prognosis.^9^ As SLE is a systemic disease, there also remains a need to identify earlier predictors of overall SLE response as well as LN responses in this population.

The Aspreva Lupus Management Study (ALMS) was a prospective, randomised, open-label, parallel group, multi-centre clinical trial that compared mycophenolate mofetil (MMF) to intravenous cyclophosphamide (CYC) as induction for patients with LN.^2^ Three hundred and seventy patients with SLE^10^ with class III–V LN were randomised to receive MMF (target dose 3 g/d) or CYC (0.5 to 1.0 g/m^2^ in monthly pulses) for 24 weeks. The primary endpoint was defined as a decrease in urine protein/creatinine ratio (P/Cr), calculated from a 24-hour urine collection, to <3 g/g in patients with baseline nephrotic range P/Cr (≥3 g/g), or by ≥50% in patients with subnephrotic baseline P/Cr (<3 g/g), and stabilisation (±25%) or improvement in serum creatinine at 24 weeks as adjudicated by a blinded Clinical Endpoints Committee. MMF was deemed non-superior for induction treatment in LN,^2^ with similar renal and non-renal response rates for both MMF (56.2%) and CYC (53%).^11^ The ALMS maintenance trial subsequently randomised those patients that responded to the induction phase to either MMF (2 g/d) or azathioprine (AZA) (2 mg/kg/d) with a follow-up period of 36 months. The cumulative probability or remaining free of treatment failure was significantly higher in the MMF group compared with the AZA group.^12^

Secondary analysis to date of the ALMS induction trial has provided further insights. Black and Hispanic patients were less likely to respond to CYC compared with MMF^13^ and non-Hispanic ethnicity was associated with a higher likelihood of complete renal response (CRR) (OR=2.0).^13^ Baseline predictors of renal response at 6 months identified included estimated glomerular filtration rate (eGFR), complement C4 and time since LN diagnosis. A rapid decline in proteinuria (>25%) within the first 8 weeks and early restoration of normal complement levels also predicted response (regardless of treatment group).^14^ Response rates in those with poor renal function (eGFR <30) were similar (MMF (20%) vs CYC (16.7%)), but patients with this level of renal impairment may have responded faster to MMF.^15^ CYC and MMF were equally efficacious for non-renal disease.^11^

MASTERPLANS is an MRC-funded consortium, whose aim is to identify predictors of treatment response in SLE. Using data from the ALMS trials, we aimed to identify clinical predictors of lupus response overall in the ALMS trial population using outcomes based on the ‘classic’ British Isles Lupus Assessment Group (BILAG) Index scoring system. We also aimed to ascertain whether predictors of renal response were different from the predictors of the overall lupus response and if any interactions with treatment use were evident.

## Methods

Baseline data collected in the ALMS induction and maintenance trials were used for this post hoc analysis of predictors of response at 6 months. As the original trial found that the MMF and CYC arms had relative homogeneity in terms of baseline demographics and response rates, the whole trial population was analysed as a single cohort.

The BILAG-based endpoints at 6 months were;

1. Major Clinical Response (MCR): Reduction in BILAG score to BILAG C in all domains, a reduction in steroid dose to ≤7.5 mg daily and a Systemic Lupus Erythematosus Disease Activity (SLEDAI) score ≤4. We note that the trial protocol did not mandate (but did permit at the physician‘s discretion) steroid reductions to any pre-specified target.
2. Improvement: Reduction in BILAG score to no more than one BILAG B and no new BILAG organ domains involved, no increase in steroids from baseline and no increase in SLEDAI from baseline.
3. Complete Renal Response (CRR): BILAG A or B in the renal domain and a 24-hour urinary protein >500 mg/day and/or urine P/Cr >50 mg/mmol and/or urine albumin/creatinine ratio >50 mg/mmol at baseline, with follow-up urine P/Cr ≤50 mg/mmol and eGFR) using the Modification of Diet in Renal Disease (MDRD) formula ≥60 ml/min/1.72 m^2^ OR if eGFR ≤60 ml/min at baseline, eGFR to not have fallen by ≥20% compared with the baseline value.
4. Partial Renal Response (PRR): BILAG A or B in the renal domain and a 24-hour urinary protein >500 mg/day and/or urine P/Cr >50 mg/mmol and/or urine albumin/creatinine ratio >50 mg/mmol at baseline, with follow-up urine P/Cr ≤100 mg/mmol and eGFR ≥60 ml/min OR if eGFR ≤60 ml/min at baseline, eGFR to not have fallen by ≥20% compared with the baseline value. By definition, CRR patients also are within the PRR subset.

Descriptive statistics were used to summarise enrolment data at the baseline.^2^ Univariate logistic regression was used to calculate ORs of the following potential predictors of response derived from previous literature and clinical expert agreement;

1. Demographics: gender, race/ethnicity, geographical region, age at enrolment, height, weight, LN duration.
2. Concomitant medications: Angiotensin Converting Enzyme (ACE) inhibitors, diuretics, aminoquinolines (antimalarials), calcium, calcium with others, dihydropyridine (calcium channel blockers), H2 receptor antagonists, proton pump inhibitor, sulfonamides, vitamin D, steroids dose.
3. Comorbidities: diabetes and hypertension.
4. Laboratory parameters: lupus anticoagulant, ANA, anti-dsDNA antibodies, anticardiolipin IgM and IgG antibodies, C3, C4 and CH50 levels, haemoglobin, differential lymphocyte count, differential neutrophil count, platelet count total, immunoglobulins IgG and IgM, serum albumin, baseline eGFR and 24-hour urine protein.
5. Disease activity and damage: SLEDAI, Systemic Lupus International Collaborating Clinics/American College of Rheumatology (SLICC/ACR) damage index (SDI),^16^ Classic BILAG index.^17^

Asian ethnicity and the Asian region were chosen as the reference groups as they were the biggest populations within the ALMS trial. For steroid dose and SDI scores, we created a separate category for missing data ('not available' - NA). For all other categorical preditors, we combined missing data with the reference categories. This is to retain as many samples as possible for building prediction models. We also examined the interactions between each predictor and the treatment received in the above univariate logistic regressions.

Logistic regressions with shrinkage estimators, that is, least absolute shrinkage and selection operator (LASSO) and elastic net, were used to build multivariate prediction models.^18^ Tenfold cross-validation with 500 times of repeated random splitting was used; in total 5000 prediction models were built. Each model used a training subsample of the data (ninefolds in a specific data split), where the tuning parameters of LASSO and elastic net were selected by cross-validation. Predicted probabilities for the testing samples in the remaining fold were calculated. The predicted probabilities were then averaged across 500 replications (due to repeated random splitting) to generate a final predicted probability for each sample. The prediction performance of the models was summarised by area under the receiver operating characteristic (ROC) curves, that is, AUROC. We ranked the predictors by their frequencies of being chosen by LASSO among the 5000 models to provide an indication of the importance of the predictors. Additionally, random forests were used to check if there were interactions and non-linearity among the variables selected by LASSO.^19^ The analysis was conducted using SAS University edition and R.

## Results

Three hundred and seventy patients were enrolled in the ALMS induction trial.^2^ Baseline patient demographics are detailed in table 1, along with baseline disease characteristics in table 2.

**Table 1:** Baseline patient demographics in the induction phase of the ALMS trial

Characteristic	Total (N= 370)
Gender (n (%))	
Male	57 (15.41)
Female	313 (84.59)
Race (n (%))	
Caucasian	147 (39.73)
Asian	123 (33.24)
Black	46 (12.43)
Other races	54 (14.59)
Region (n (%))	
Asia	117 (31.62)
Latin America (LA)	106 (28.65)
Europe	61 (16.49)
USA/Canada	75 (20.27)
Rest of the world	11 (2.97)
Race/location (n (%))	
Asian	123 (33.24)
Black in other regions	15 (4.05)
Black in the USA	31 (8.38)
Caucasian in Europe	51 (13.78)
Caucasian in Latin America and other regions	66 (17.84)
Caucasian in USA/Canada	30 (8.11)
Other races	54 (14.59)
Age at baseline (years; mean±SD)	31.8±10.6
Disease duration of lupus nephritis (years; median (range))	1.0 (1 to 23)
Disease duration of lupus nephritis, by category (n (%))	
≤1 year	236 (63.78)
2–4 years	69 (18.65)
>4 years	65 (17.57)
Height (m; mean±SD)	1.62±0.09
Weight (kg; mean±SD)	64.21±15.12
Diabetes (n (%))	6 (1.62)
Hypertension (n (%))	202 (54.59)
Steroid dose (prednisone mg/day) (n (%))	
<50	91 (24.59)
50–60	94 (25.41)
>60	175 (47.30)
NA	10 (2.70)

ALMS, Aspreva Lupus Management Study.

**Table 2:** Baseline disease characteristics in the induction phase of the ALMS trial

Characteristic	Total (N= 370)
Anti-dsDNA (n (%))	
High	310 (83.78)
NA	9 (2.43)
Normal	51 (13.78)
ANA (n (%))	
High	186 (50.27)
NA	123 (33.24)
Normal	61 (16.49)
Complement C3 (n (%))	
High	2 (0.54)
Low	274 (74.05)
NA	7 (1.89)
Normal	87 (23.51)
Complement C4 (n (%))	
High	4 (1.08)
Low	215 (58.11)
NA	8 (2.16)
Normal	143 (38.65)
Haemoglobin (n (%))	
High	2 (0.54)
Low	212 (57.30)
NA	16 (4.32)
Normal	140 (37.84)
Lymphocytes (n (%))	
High	15 (4.05)
Low	74 (20.00)
NA	21 (5.68)
Normal	260 (70.27)
Platelets (n (%))	
High	43 (11.62)
Low	12 (3.24)
NA	24 (6.49)
Normal	291 (78.65)
Immunoglobulin IgG (n (%))	
High	85 (22.97)
Low	67 (18.11)
NA	1 (0.27)
Normal	217 (58.65)
Lupus anticoagulant (n (%))	
NA	14 (3.78)
Negative	309 (83.51)
Positive	47 (12.70)
Anticardiolipin IgM (n (%))	
High	25 (6.76)
NA	103 (27.84)
Normal	242 (65.41)
Anticardiolipin IgG (n (%))	
High	67 (18.11)
NA	103 (27.84)
Normal	200 (54.05)
SLICC/ACR damage index (without renal category) (n (%))	
0	195 (52.70)
1	50 (13.51)
>1	29, (7.84)
NA	96 (25.95)
SLEDAI score (mean±SD)	15.28±6.78
BILAG general A or B (n (%))	62 (16.80)
BILAG haematology A or B (n (%))	138 (37.30)
BILAG cardiorespiratory A or B (n (%))	21 (5.69)
BILAG mucocutaneous A or B (n (%))	108 (29.19)
BILAG musculoskeletal A or B (n (%))	62 (16.80)
BILAG neuropsychiatric A or B (n (%))	8 (21.62)
BILAG renal A or B (n (%))	367 (99.19)
BILAG vasculitis A or B (n (%))	19 (5.14)

ALMS, Aspreva Lupus Management Study; BILAG, British Isles Lupus Assessment Group; NA, data not available /missing; SLEDAI, Systemic Lupus Erythematosus Disease Activity; SLICC/ACR, Systemic Lupus International Collaborating Clinics/American College of Rheumatology.

### Major Clinical Response

MCR was achieved by 14 (3.79%) at 6 months. Due to the low number of patients obtaining MCR at 6 months, further analysis of this endpoint was not performed.

### Improvement

Improvement was attained by 188 (50.81%) at 6 months. Predictors included older age (OR (95% CI)=1.03 (1.01 to 1.05) per year) and normal haemoglobin (1.85 (1.16 to 2.95) vs low haemoglobin). Active disease (BILAG A or B) in haematological and mucocutaneous domains predicted less likelihood of improvement (0.61 (0.39 to 0.97) and 0.50 (0.31 to 0.81), respectively). Baseline damage (SDI>1 vs 0) (0.38 (0.16 to 0.91)) and 24-hour urine protein (0.63 (0.50 to 0.80)) also negatively predicted 6-month improvement.

### Complete Renal Response

CRR was achieved by 75 (20.27%) at 6 months. Latin American location (0.47 (0.23 to 0.94) vs Asian location), 24-hour urine protein (0.63 (0.45 to 0.89)) and LN duration 2–4 years (0.43 (0.19 to 0.97) vs <1 year) were negative predictors.

### Partial Renal Response

PRR was achieved by 198 (53.51%) at 6 months. Lupus anticoagulant positivity (0.37 (0.19 to 0.73) vs negative/NA), a normal neutrophil count (0.50 (0.28 to 0.89) vs high), calcium supplementation (0.42 (0.20 to 0.87) vs no calcium) and LN duration 2–4 years (0.45 (0.24 to 0.83) vs <1 year) were negative predictors.

**Figure 1:**
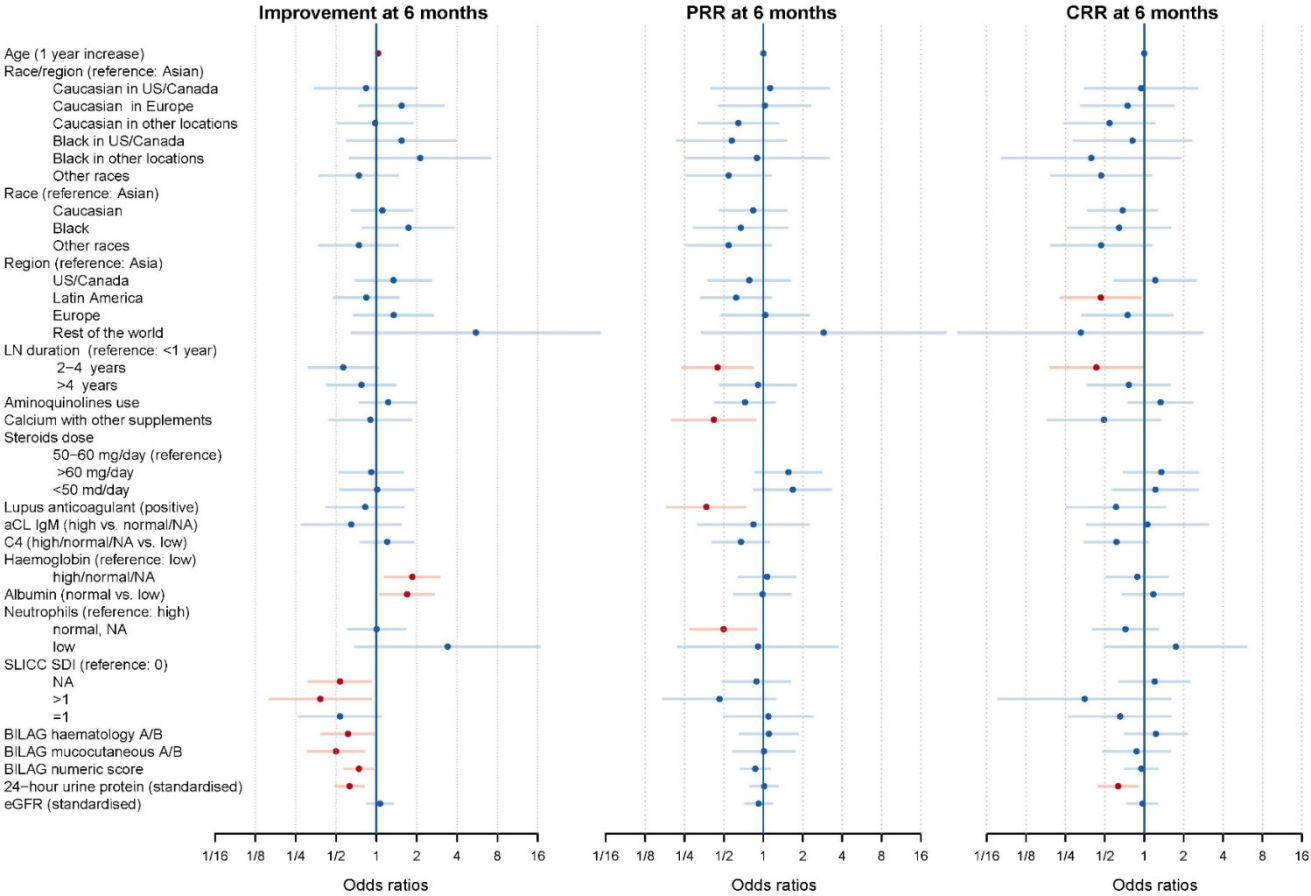
Univariate analysis of improvement, partial renal response (PRR) and complete renal response (CRR) at 6 months. Red circles/bars represent odds ratios and 95% CI of statistically significant predictors (p≤0.05) for PRR and CRR, and blue circles/bars represent ORs and 95% CI of non-significant predictors for PRR and CRR. aCL, anticardiolipin; BILAG, British Isles Lupus Assessment Group; eGFR, estimated glomerular filtration rate; LN, lupus nephritis; SDI, SLICC/ACR damage index; SLICC, Systemic Lupus International Collaborating Clinics.

Further results of the univariate analysis are demonstrated in figure 1.

### Interactions between predictors and treatments

We found no conclusive interactions between individual predictors and treatments (MMF/IVC) at the induction phase in our univariate logistic regressions.

### Multivariate predictions

AUC results for multivariate logistic regressions with LASSO and elastic net and random forests were very similar; here, we only report the results with LASSO. Specifically, the AUROCs of models for improvement, CRR, PRR at 6 months were 0.56, 0.55 and 0.51, respectively. Multivariate model results were consistent with the univariate analyses, where the above predictors identified in univariate analyses were also most often selected by LASSO. In online supplemental materials, we present the frequencies that each predictor was chosen among the 5000 prediction models in online supplemental figures 1–3.

10.1136/lupus-2021-000584.supp1 Supplementary data

10.1136/lupus-2021-000584.supp2 Supplementary data

10.1136/lupus-2021-000584.supp3 Supplementary data

## Discussion

Clinical trials in SLE are challenging and frequently fail to meet their primary endpoint for various potential reasons. Both the heterogeneity of SLE disease manifestations and the small numbers of patients available for recruitment to clinical trials may contribute to this.^20^ Trials in SLE may also be restrictive in their inclusion criteria with regard to renal disease and, as such, lack a degree of external validity.^21^ Endpoint definitions have been consistently difficult to agree on, but there is a movement towards composite disease activity scores such as the SLE Responder Index after its successful employment in the phase 3 belimumab trials.^22^ Major concerns remain, with additional ‘noise’ caused by polypharmacy and traditionally high dose steroid use within SLE populations potentially contributing to trial failure.^21^ The MASTERPLANS consortium aims to develop early clinical predictive markers in SLE to help inform future trials and personalised medicine studies. In LN trials, several traditional poor prognosis markers are enriched as these patients often have a more severe disease phenotype. Knowledge of and stratification for such markers may improve the conduct of future trials. In clinical practice, it may be possible to employ such markers to inform the treatment strategy used and to improve overall treatment response rates.

Our results found a number of predictors of global lupus and renal-specific responses which are of interest when considering treating patients with SLE and LN. Importantly, predictors of global response at 6 months tended to be different to those that predicted renal outcomes over the same period. Disease activity on BILAG and damage on SDI were associated with global outcomes but were not predictive of renal outcomes. This observation is relevant to future LN trials as balancing non-renal manifestations may influence overall outcomes since trials assess both renal and non-renal changes in their outcome assessments.

LN disease duration of 2–4 years was associated with a decreased likelihood of achieving CRR and PRR at 6 months. This has also been shown by others, with longer lupus disease duration considered a negative predictor of achieving overall low disease activity, although not specifically renal outcomes.^23^ Longer disease duration may act as a surrogate for a more relapsing-remitting course of LN and also of course may link to some early renal damage that limits a patient’s ability to achieve stringent response targets.

Patients recruited from Latin America had a decreased likelihood of attaining CRR at 6 months compared with our Asian comparator group. Studies have consistently shown that patients from Hispanic backgrounds develop LN early and have more aggressive disease.^24 25^ This could be explained by socioeconomic factors and variable access to healthcare within the regions, however in a trial setting more consistent provision of therapy would tend to mitigate this. Latin America itself is very ethnically diverse with Caucasians, Mestizo, pure Amerindians and African-Latin Americans all recognised ethnic subgroups.^26^ Such consistent findings across outcomes do suggest that a complex interaction of factors influence LN outcomes in this region. Our study however lacked power to dissect this out in more detail. While Asian ethnicity is also diverse and is traditionally associated with severe renal disease,^27^ their response to treatment, long-term renal outcomes and renal survival rates appear to be better, particularly when compared with Hispanic populations.^28^ In the SLICC inception cohort, we previously found that Asian patients (from South Korea) had less progression to damage over time.^29^ These results point to potential organ-specific differences in responsiveness among patients from different racial and ethnic backgrounds. The potential prognostic role of ethnicity has also been considered previously in the literature comparing ALMS maintenance and MAINTAIN nephritis trials. Both trials assessed the efficacy of MMF for maintenance therapy, with the former suggesting MMF as superior for the treatment of LN and the latter suggesting no difference. MAINTAIN was a European study with a predominantly Caucasian population, whereas ALMS was an international study with more ethnic diversity (79% and 44% Caucasian, respectively).^30^ The superiority of MMF in the ALMS study may at least be partially explained by the ethnic background of those enrolled.^31^

Established damage at baseline was associated with a decreased likelihood of achieving global improvement by 6 months. Higher SDI scores at baseline increase the risk of mortality in patients with SLE.^29^ Established damage will reflect more severe previous disease and/ or higher chronic steroid exposure and will also be more prevalent in patients with longer disease duration. Activity (BILAG A or B) in haematological and mucocutaneous domains predicted less improvement which supports findings in the EXPLORER trial, where baseline BILAG mucocutaneous involvement was not predictive of treatment response.^32 33^

Considering haematological involvement, baseline BILAG A or B scores have been demonstrated to predict flares at 24 and 52 weeks^34^ in the phase III belimumab trials. While the endpoints in this analysis were different, those patients who are going to flare would be less likely to achieve improvement. Patients with higher baseline disease activity are also likely to be harder to treat and may require different therapeutic strategies. An increase in the numerical BILAG was also associated with a decreased likelihood of improvement at 6 months so overall more extensive disease even when using potent immunosuppression in LN is associated with poorer response rates. The ALMS induction trial^2^ has reported previously the efficacy of MMF and CYC in achieving good BILAG non-renal responses, with particularly promising improvement in BILAG index scores within the mucocutaneous (MMF 84% vs CYC 93%) and musculoskeletal (MMF 91% vs CYC 96%) at 24 weeks.^11^ This research was evaluating individual disease activity in individual systems but we have demonstrated when considering the patient overall, it is harder to achieve composite non-renal outcomes with only 50.81% achieving improvement at 6 months.

A previous study using this dataset found very few multivariate baseline predictors of renal response and/or renal remission.^3^ In contrast to the study by Dall’Era *et al*, the current study was focused on BILAG-based outcomes in this trial and assessed renal responses as well as overall SLE responses. Also, in contrast to Dall’Era *et al*,^3^ our renal endpoints of MCR and PRR did not set different response criteria based on whether the patient was nephrotic or not at baseline. Also comparing the ‘renal response’ definition to our equivalent PRR, we used a lower absolute value of urine P/Cr ratio of <100 mg/mmol rather than percentage reduction in proteinuria for subnephrotic patients. Our study therefore complements and adds to this previous analysis by also including overall SLE responses within the trial, which means we were also able to compare and contrast the factors that predict renal and overall SLE responses to show different factors associated with each.

### Limitations

ALMS was considered a large global trial at its time but a sample size of 370 still limits our power to identify all important predictors of response in SLE. Trials with larger populations would provide more precision to predictor estimates. We focused on 6-month outcomes in this analysis and while 12-month data was available it was only available for those who showed a level of response at 6 months and that qualified them for re-randomisation. Data beyond 6 months for those not re-randomised was therefore not available.

The predictive performance of the clinical model examined, as shown by the AUROC results, was very modest and implies that any model combing these baseline factors will have a poor ability to predict treatment response. Our variable selection results do however show the relative predictive power of each factor compared with each other and help identify patient characteristics who respond better to conventional therapies. Taken together, our results emphasise the need to identify novel biomarkers that will improve the predictive accuracy for treatment response in patients with SLE over and above the modest performance of clinical factors alone. Urinary biomarkers have recently been demonstrated to predict treatment response to rituximab in LN at 6 months.^35^ Adding such factors into our models would likely further improve their predictive value. Continuing to identify such biomarkers remains the long-term aim of the MASTERPLANS Consortium.

## Conclusion

We have identified a number of baseline clinical variables that predict outcomes at 6 months in patients with active SLE/LN.

Different variables tended to predict renal and non-renal outcomes, with LN disease duration associated with renal outcomes and more active extra-renal disease with global responses. Such factors should be considered and balanced in future SLE trials and outcome studies and may also identify patients who will need alternative treatment strategies to conventional immunosuppressive agents.

## Data Availability

Data are available upon reasonable request.

## Data availability statement

Data are available upon reasonable request to the MASTERPLANS consortium through the corresponding author.
