# Supplementary material for: Predictors of treatment response in a lupus nephritis population: lessons from the Aspreva Lupus Management Study (ALMS) trial
Source: Lupus Sci Med. 2022 May 30;9(1):e000584. doi: 10.1136/lupus-2021-000584 (PMC9157342; doi:10.1136/lupus-2021-000584)
Supplement: Supplementary data [file lupus-2021-000584supp002.pdf]

Supplementary Figure S2: Partial Renal Response at 6 months

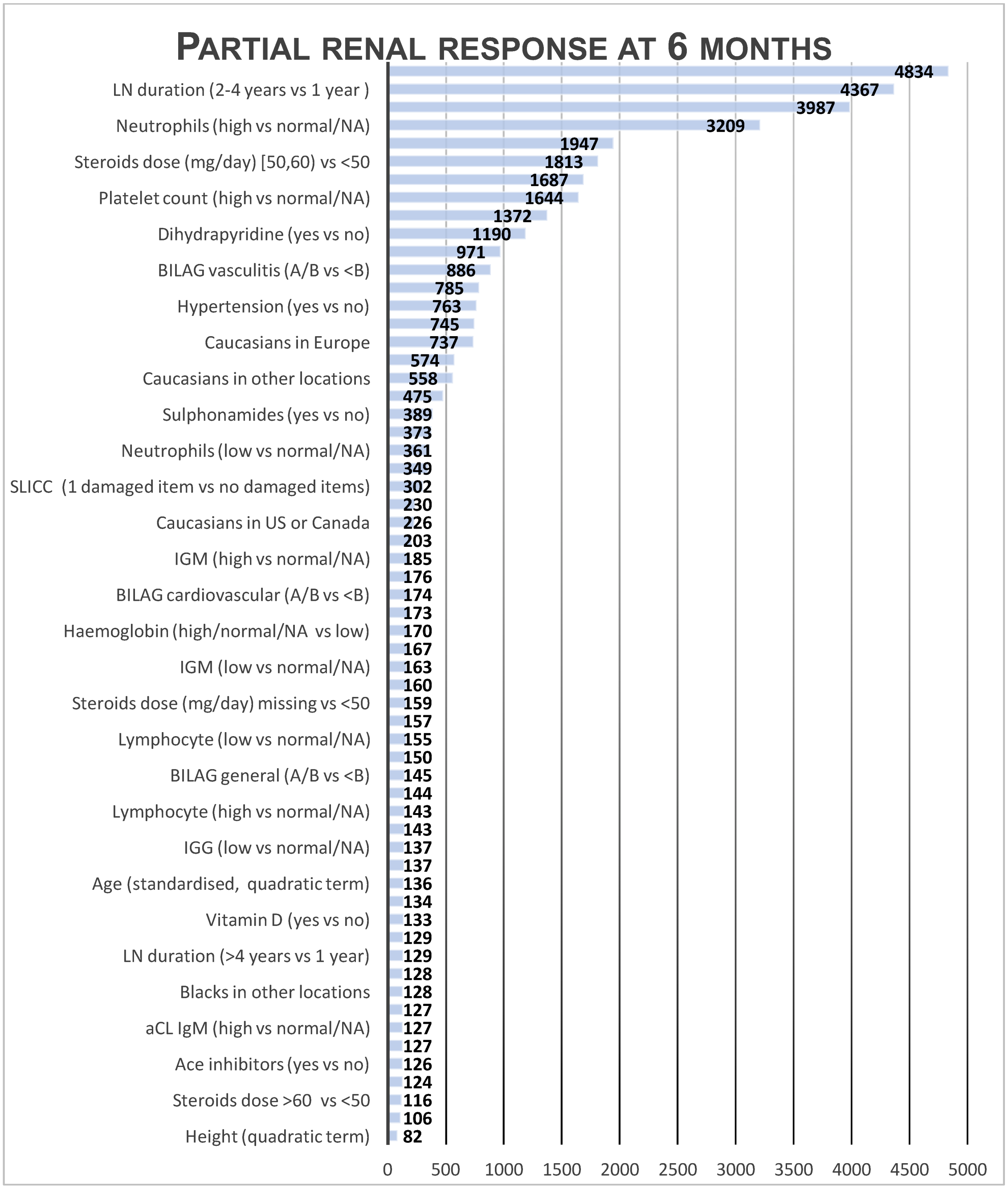

Blue bars represent frequencies that each predictor was chosen by LASSO
